# Alleviation of NaCl Stress on Growth and Biochemical Traits of *Cenchrus ciliaris* L. via Arbuscular Mycorrhizal Fungi Symbiosis

**DOI:** 10.3390/life14101276

**Published:** 2024-10-08

**Authors:** Jahangir A. Malik, Abdulaziz A. Alqarawi, Fahad Alotaibi, Muhammad M. Habib, Salah N. Sorrori, Majed B. R. Almutairi, Basharat A. Dar

**Affiliations:** 1 Plant Production Department, College of Food & Agriculture Sciences, King Saud University, P.O. Box 2460, Riyadh 11451, Saudi Arabia; jmalik@ksu.edu.sa (J.A.M.); alqarawi@ksu.edu.sa (A.A.A.); mhabib@ksu.edu.sa (M.M.H.); salahsar2008@gmail.com (S.N.S.); 2 Department of Soil Science, College of Food & Agriculture Sciences, King Saud University, P.O. Box 2460, Riyadh 11451, Saudi Arabia; fanalotaibi@ksu.edu.sa (F.A.); m3aden2010@hotmail.com (M.B.R.A.)

**Keywords:** growth, arbuscular mycorrhizal fungi (AMF), salinity, *Cenchrus ciliaris*

## Abstract

Soil salinization, especially in arid and semi-arid regions, is one of the major abiotic stresses that affect plant growth. To mediate and boost plant tolerance against this abiotic stress, arbuscular mycorrhizal fungi (AMF) symbiosis is commonly thought to be an effective tool. So, the main purpose of this study was to estimate the role of AMF (applied as a consortium of *Claroideoglomus etunicatum*, *Funneliformis mosseae*, *Rhizophagus fasciculatum*, and *R. intraradices* species) symbiosis in mitigating deleterious salt stress effects on the growth parameters (shoot length (SL), root length (RL), shoot dry weight (SDW), root dry weight (RDW), root surface area (RSA), total root length (TRL), root volume (RV), root diameter (RD), number of nodes and leaves) of *Cenchrus ciliaris* L. plants through improved accumulations of photosynthetic pigments (chlorophyll *_a_*, chlorophyll *_b_*, total chlorophyll), proline and phenolic compounds. The results of this experiment revealed that the roots of *C. ciliaris* plants were colonized by AMF under all the applied salinity levels (0, 75, 150, 225, and 300 mM NaCl). However, the rate of colonization was negatively affected by increasing salinity as depicted by the varied colonization structures (mycelium, vesicles, arbuscules and spores) which were highest under non-saline conditions. This association of AMF induced an increase in the growth parameters of the plant which were reduced by salinity stress. The improved shoot/root indices are likely due to enhanced photosynthetic activities as the AMF-treated plants showed increased accumulation of pigments (chlorophyll *_a_*, chlorophyll *_b_* and total chlorophyll), under saline as well as non-saline conditions, compared to non-AMF (N-AMF) plants. Furthermore, the AMF-treated plants also exhibited enhanced accumulation of proline and phenolic compounds. These accumulated metabolites act as protective measures under salinity stress, hence explaining the improved photosynthetic and growth parameters of the plants. These results suggest that AMF could be a good tool for the restoration of salt-affected habitats. However, more research is needed to check the true efficacy of different AMF inoculants under field conditions.

## 1. Introduction

Plant species naturally face various abiotic stresses (e.g., salinity) that can reduce the productivity and growth of plants [1]. Worldwide, mainly in semi-arid and arid areas, plants deal with salinity stress, which is believed to be one of the most disturbing abiotic stresses [2,3]. Soil salinity (ECe > 4 dSm^−1^) affects around 10% of the total arable land [4] and more than a billion hectares in total [5]. Natural processes produce excessive salt concentrations in the soil, and because of human impacts, they become aggravated (10%/year) [6]. In the 21st century, saline soils are increasing [7], and it is estimated that salinity will affect 50% of the earth’s cultivable land by the midpoint of this century [8].

Under salinity stress, plants undergo various aberrant biochemical, physiological, and morphological variations that result in poor and inhibited growth, lower yields, delayed germination, and high seedling mortality [9,10]. It also damages the growth and function of the plant by decreasing water uptake capacity, damaging the structure of the root, causing ionic toxicity, and producing osmotic stress [11].

In the past few years, the responses of plants against stress have been investigated widely, and the role of plant-microbe interactions in these responses has received paramount attention in recent years. The use of soil-borne microorganisms, like AMF, is one of the most significant approaches that have been developed to decrease soil salinization and increasing plant resilience in saline environments [12]. AMF, belonging to the phylum *Mucoromycota*, are widespread soil fungi that form obligate symbiotic associations with 80–90% of terrestrial plants [13,14]. They enhance nutrients as well as the water uptake capacity of the plants via root surface area expansion and, in return, get fundamental lipids and carbohydrates from the plants [15]. In saline habitats, they naturally co-exist with halophyte roots and colonize 80% of the terrestrial plants [16]. 

In comparison to non-AMF (N-AMF), AMF symbiosis could improve the plant’s potential to withstand salt stress by improving the nutrient uptake efficiency [17], producing plant growth hormones [18], enhancing rhizospheric soil [19], accumulation of compatible osmolytes and ion balance [20], increasing photosynthetic activity as well as water use efficiency [21], and proline and phenolic accumulation [2,22]. In previous studies, it was found that several factors (mycorrhizal partners, host types, salinity levels, and environmental circumstances) cause variations in the AMF impacts on plant tolerance against salinity [23,24,25]. Understanding the significance of these factors in AM symbiosis with plants is essential to explain the AMF-mediated plant salinity tolerance mechanism.

Buffelgrass (*Cenchrus ciliaris* L.) is a member of the family Poaceae [26]. It is a drought-resistant [27], nutritiously valuable [28], perennial, C4, and halophytic forage grass [29], which mainly grows on marginally fertile shallow soils [30]. This plant is indigenous to the hot and dry areas of Australia, Asia, Africa [31], and the Arabian Peninsula [27]. *C. ciliaris* is widely grown and well adapted to arid rangelands of Saudi Arabia and can withstand heavy grazing [32], which makes it the best candidate for both forage as well as rangeland restoration and stabilization [33,34].

*C. ciliaris* is widely acknowledged for its association with AMF [35], as prior studies have stated that AMF symbiosis promotes the nutrient intake, water use efficiency, chlorophyll content, yield, and overall growth of the plant [36,37,38]. However, to our knowledge, no similar study has been conducted to explore the AMF symbiosis potential in improving the performance of *C. ciliaris* under saline conditions in Saudi Arabia. So, the core aim of the present research was to study the role of AMF symbiosis in alleviating the salinity stress on *C. ciliaris*.

We hypothesized that: (1) the growth of *C. ciliaris* might be negatively affected by salinity stress as it changes the physiological and biochemical activities; (2) salinity imposition levels and duration change the effects of AMF on the growth of plants; (3) the plant’s resistance against salinity will improve because of increasing phenolic compounds and proline production

We will gain a better understanding of AMF symbiosis’ role in enabling desert flora to adapt to high salinity from this study, making it a simple and alternative method for regenerating ecosystems that have been negatively impacted by salinity stress.

## 2. Materials and Methods

### 2.1. AMF Inoculum

The AMF inoculum used in the present experiment was originally derived from the rhizosphere of naturally growing *C. ciliaris* L. in the Al-Ghat (26°02′86.1″ N; 44°54′74.7″ E) region of Saudi Arabia. The AMF assemblages obtained from the collected rhizospheric soil before being used as inoculum were propagated in a trap culture with sorghum bicolor as a host plant for four months. The trap culture soil was later assessed for AMF spores via the wet sieving and decanting method [39] and the spores were identified by following the previously available literature [40,41] and INVAM [42] (accessed on 24 March 2024). A mixture of AMF species, viz., *Claroideoglomus etunicatum*, *Funneliformis mosseae*, *Rhizophagus fasciculatum*, and *R. intraradices* were identified from the trap culture and used as inoculum with a density of ~200 spores g^−1^ of dry soil. The inoculum soil also contained colonized sorghum roots.

### 2.2. Seeds and Soil Preparation

The seeds used in this experiment were collected from fully grown and mature spikes of *C. ciliaris* plants in Al-Ghat, Riyadh region, Saudi Arabia (26°02′86.1″ N; 44°54′74.7″ E), where it grows naturally. The collected seeds were transported to the Range Science, KSU laboratory, where they were air-dried at room temperature and stored for further experimentation. Sandy loam soil, which was obtained from the same habitat/region as the target seeds, was selected to simulate the natural habitats of *C. ciliaris*. The physico-chemical properties of the soil are presented in Table 1. Prior to filling the experimental pots, the soil was autoclaved at 123 °C for 120 min to eliminate microbial contamination and set the base for AMF inoculation. Subsequently, the soil was dried, crushed, and sieved through 8 mm mesh.

### 2.3. Experimental Design

The experiment was set under controlled conditions in the growth chamber of the plant production department at King Saud University, Riyadh, Saudi Arabia. The average temperature was maintained at 35/25 °C (D/N) with a light duration of 14/10 h (L/D) throughout the experimental period. The relative humidity was maintained within the range of 50–60%. The dried seeds of *C. ciliaris* with fully mature grains were selected, and surface sterilized in 70% ethanol for 5 min followed by sodium hypochlorite treatment for 10 min. The seeds were then rinsed with sterile distilled water and left soaked in distilled water for 24 h under dark conditions. Next, five seeds were sown in each experimental pot (top diameter: 14 cm, height: 13 cm, and bottom diameter: 11 cm) filled with 1 kg of dried, autoclaved sandy loam soil. The soil was amended with 5% AMF inoculum. For the N-AMF treatments, the plants were provided an equal quantity of sterilized AMF inoculum (autoclaved at 123 °C for 120 min) along with a filtrate (<20 µm) from the AMF inoculum to maintain a general microbial population devoid of AMF spores. This allowed a controlled comparison between AMF and N-AMF. The seedlings, once fully established, were thinned to two seedlings per pot to avoid competition. The salinity was applied after one month of the experiment to allow the better establishment of AM colonization and plant growth. Pots were irrigated as and when required to avoid drought stress. At every irrigation, the electrical conductivity (EC) of the pot soil was routinely tested with an EDT NE287 Micro Conductivity Meter (Hanna instruments, Woonsocket, RL, USA) to ensure the salinity treatments were maintained for the whole period of experimentation.

The experiment was conducted in a complete randomized design (CRD) with two factors. The AMF inoculation with two levels, viz., (1) AMF and (2) N-AMF, were treated as one factor, and five salinity stress levels (0 mM NaCl (Control), 75 mM NaCl, 150 mM NaCl, 225 mM NaCl, 300 mM NaCl) were considered as the second factor. Each treatment was repeated five times so a total of (2 × 5 × 5) = 50 pots with five seeds each were arranged randomly.

Following ninety days of NaCl application i.e., four months of plant growth, the plants reflected the signs of wilting at high NaCl concentrations. Therefore, during this period we harvested the experiment and collected the samples for different measurements.

### 2.4. AMF Colonization Rate and Spore Count Estimation in the Roots of C. ciliaris

Fifty random root segments were selected from each treatment for the study of mycorrhizal colonization. The fine roots were picked, sorted, and cleaned in distilled water with care. The washed roots were then boiled with 10% KOH in a water bath for 30 min at 80 °C, washed again in distilled water, and passed through H_2_O_2_ (3%) for 3 min before being acidified with 1% HCl for 10 min and then stained in Trypan blue for another 20 min at 80 °C [43]. Later, the segments were sliced and mounted on slides using lactoglycerol solution and quantified for different colonization structures (mycelium, vesicles and arbuscules) under a 10 × 40 magnification microscope [44]. The roots of plants treated with N-AMF inoculum were also studied and showed no colonization structures.

The spores were extracted from the substrate of each treatment using the methods described previously [39]. The total spore population in each treatment was determined from 100 g of dry soil sample.

### 2.5. Estimation of Morphological Parameters

From each treatment, *C. ciliaris* plants were harvested and separated into shoot and root systems for analysis. The roots were cleaned with tap water tape water and surface water was dried using filter paper. Later, the shoot and root lengths were measured, and part of the roots were scanned for measurement of additional root parameters such as root surface area (RSA), total root length (TRL), root volume (RV) and root diameter (RD) via WinRHIZO software (v5.0, Regent Instruments, Quebec, QC, Canada). For the shoot (SDW) and root dry weight (RDW), the samples were dried in an oven at 75 °C for 48 h. Prior to biomass estimation, the plants were studied for number of nodes and leaves.

### 2.6. Chlorophyll Contents

Photosynthetic pigments (Chlorophyll *_a_*, chlorophyll *_b_*, and total chlorophyll) were quantified by following the established method of Arnon (1949) [45]. For this purpose, 100 mg of fresh plant leaves were digested in 10 mL of 80% acetone solution. The homogenate was centrifuged at 5000 rpm for 5 min with a Benchtop Centrifuge-5810R (Eppendorf, Hamburg, Germany). The samples were then incubated in the dark for three hours before being tested for absorbance at wavelengths 645 and 663 with a UV-VIS spectrophotometer (SHIMADZU, Kyoto, Japan, UV1800).

### 2.7. Estimation of Proline in C. ciliaris

To evaluate proline concentration (µg/gFW) in shoots and roots of *C. ciliaris*, 100 mg of fresh shoot and root tissue were homogenized in 3% sulfosalicylic acid (10 mL) using a mortar and pestle. The homogenate was then centrifuged at 5000 rpm for 10 min (Benchtop Centrifuge-5810R, Eppendorf, Hamburg, Germany), and 2 mL of the supernatant was transferred to a separate test tube. This extract (2 mL) was incubated for 1 h in a boiling water bath (94–100 °C) with 2 mL of glacial acetic acid and ninhydrin, then subjected to an immediate ice shock. Then, 4 mL of toluene was added, and the chromophore-containing toluene was collected into a separate tube after 20 s of mixing. Finally, the absorbance at 520 nm was measured with a UV-VIS spectrophotometer (SHIMADZU, Kyoto, Japan, UV1800) [46]. A standard curve was obtained using known proline concentrations.

### 2.8. Total Phenolic Content Estimation in C. ciliaris

By using the Folin–Ciocalteu reagent, the total phenolic activity of the shoot and root of *C. ciliaris* was estimated according to Ainsworth’s technique [47]. A total of 2 mL Folin–Ciocalteu reagent (diluted 1:10 with de-ionized water) and 4 mL aqueous Na_2_CO_3_ (7.5 percent *w*/*v*) were mixed with the sample extract (0.5 mL of 100 g/mL). A UV-VIS spectrophotometer (SHIMADZU, Kyoto, Japan, UV1800) was used to measure the reaction mixture’s absorbance at 765 nm after it had been incubated at room temperature for 30 min. To determine the total phenolic content, a linear equation of a standard curve was obtained via gallic acid. The total phenolic content value was expressed as milligrams of gallic acid equivalents per gram of dry weight (mg GAE/g DW).

### 2.9. Statistical Analysis

With the help of SAS^®^ 9.2 Software, the data were statistically evaluated. To determine whether AMF symbiosis affects diverse salinity stress tolerance levels, a two-way ANOVA was used (salt stress levels × several AMF species types). Also, Tukey (Tukey’s honestly significant difference (HSD)) tests (*p* = 0.05) were used to evaluate the impacts between treatment means when there were significant interactions among various AMF types and salt stress levels.

## 3. Results

At the end of the experiment, both status and salinity and their interaction significantly affected all the morphological and biochemical characteristics of root and shoot. However, the rate of colonization structures of *C. ciliaris*, except for shoot and root phenolic content (SLA), showed no significance for AMF status (Table 2 and Table 3).

### 3.1. Impact of Inoculation with AMF in a Consortium on the Rate of Colonization Structures in the Roots of C. ciliaris under Salt Stress

**Figure 1:**
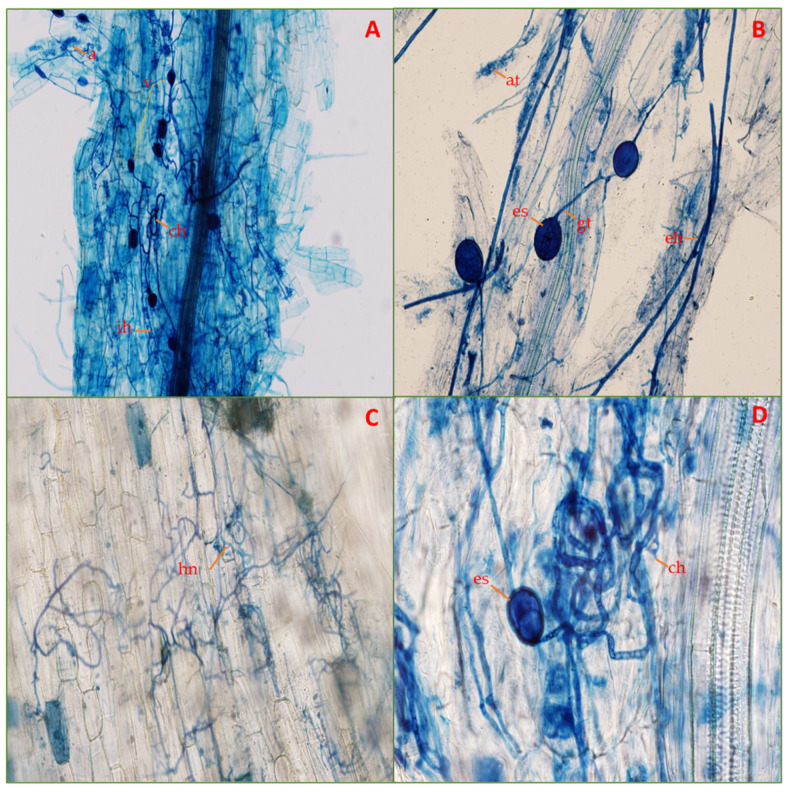
(**A**–**D**): The photomicrographs of colonized *C. ciliaris* roots taken under a microscope (10 × 40 magnification) show the perfect abundance of AMF colonization under different levels of salinity treatments. Perfect colonization is evident by the presence of: (v) vesicles; (a) arbuscules; (ch) coiled hyphae; (ih) interradical hyphae; (at) arbuscular trunk; (es) extra-radical spore; (eh) extraradical hyphae; (gt) germinating tube; and (hn) hyphal network.

**Figure 2:**
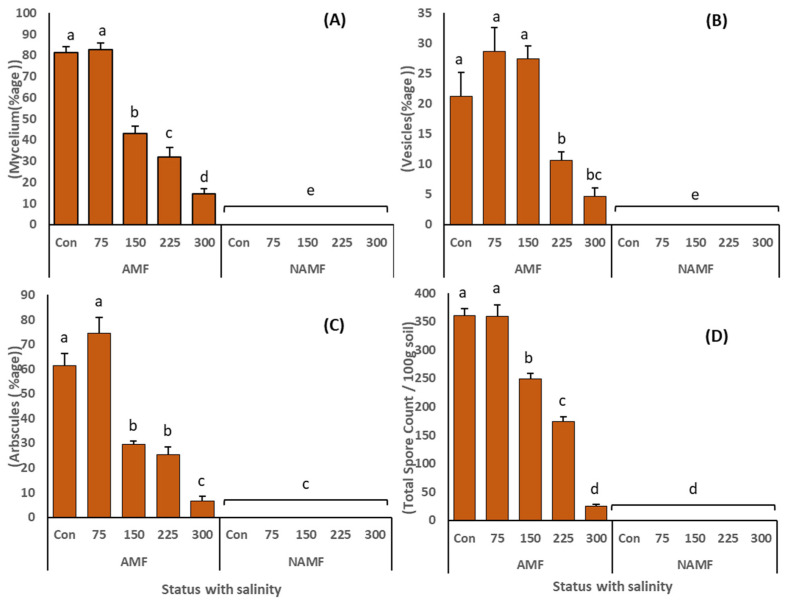
(**A**,**B**): AMF colonization rate vis-à-vis (**A**) mycelium, (**B**) vesicles and (**C**) arbuscules in the roots of *C. ciliaris* and (**D**) total spore count in the rhizosphere soil. The results are presented as a mean ± standard deviation of five replicates. Different letters on top of the bars indicate the significant difference in treatments *p* = 0.05 (Tukey’s HSD test).

A microscopic examination of the mycorrhizal status of *C. ciliaris* plants under salt stress after a cultivation period of four months revealed that all AMF-treated plants exhibited all the colonization structures (mycelium, vesicles, arbuscules, and spores) (Figure 1). However, the colonization rate was significantly reduced with the increasing NaCl stress (Figure 2, Table 3). The highest colonization rate was recorded in the plants growing under control (0 mM NaCl) or mild (75 mM NaCl) salinity stress with mycelial growth of 81.4 ± 2.6% and 82.6 ± 3.3%, respectively. At the extreme salinity stress of 300 mM NaCl, the mycelial growth was reduced to 14.6 ± 2.5%. Compared to the control (21.2 ± 3.9% vesicles), the vesicle production in roots showed a promising increase at increasing salinity levels from 75 mM (28.6 ± 3.9%) and 150 mM (27.4 ± 2.1%) which then declined abruptly at the highest salinity stress (Figure 2B). The formation of arbuscules was again altered by the salinity stress. The highest arbuscules production (74.4 ± 6.4%) was recorded in the roots of plants at 75 mM NaCl which was 1.2 times more than in the plants under no salinity stress (Figure 2C). Similar results to that of mycelial growth were displayed for the total spore count, with the highest number of spores extracted from the rhizosphere soil of plants grown at control (361.2 ± 11.7 spores/100 g dry soil) and 75 mM NaCl stress (359.8 ± 19.9 spores/100 g dry soil) (Figure 2D). The studied N-AMF roots showed no colonization.

### 3.2. Impact of Inoculation with AMF in Consortium on Morphological Parameters of C. ciliaris Grown under Salt Stress

**Figure 3:**
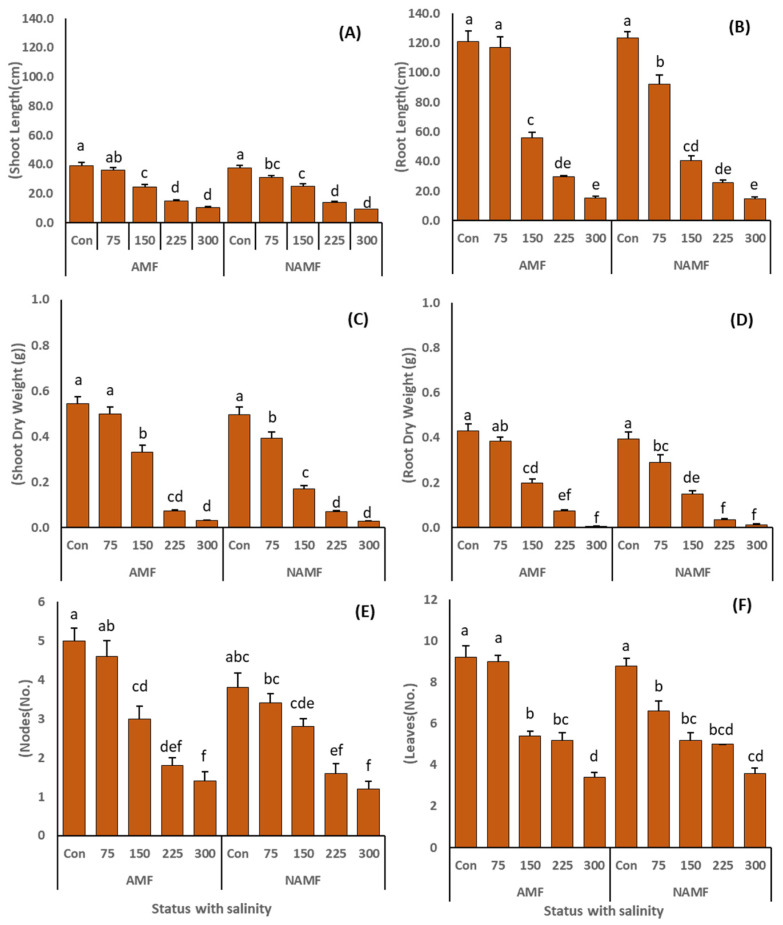
(**A**–**F**): indicates the role of AMF application as consortium (*C. etunicatum*, *F. mosseae*, *R. fasciculatum*, and *R. intraradices*) on vegetative growth factors such as (**A**) shoot length (SL), (**B**) root length (RL), (**C**) shoot dry weight (SDW), (**D**) root dry weight (RDW), (**E**) nodes (no/plant) and (**F**) leaves (no/plant) of *C. ciliaris* plants grown under salinity stress. The results are presented as a mean ± standard error of five replicates. Different letters on top of the bars indicate the significant difference in treatments *p* = 0.05 (Tukey’s HSD test).

The application of salinity led to the significant decline of morphological parameters such as SL, RL, SDW, RDW, RSA, TRL, RV, RD, number of nodes and leaves (Figure 3, Table 2). However, inoculation of plants with the consortium of AMF improved all the parameters compared to N-AMF-treated plants. The SL and RL showed a slight improvement in AMF-amended plants compared to N-AMF-inoculated plants at all salinity levels (Figure 3). However, in the case of RL, the AMF inoculation induced a sharp increase in the plants at 75 mM NaCl compared to the N-AMF-amended plants at the same salinity stress. The AMF inoculation significantly improved the SDW and RDW at the first three levels of salinity (0 mM—SDW = 0.54 ± 0.03 g; RDW = 0.43 ± 0.03 g, 75 mM SDW = 0.49 ± 0.03 g; RDW = 0.38 ± 0.01 g and 150 mM—SDW = 0.33 ± 0.018 g; RDW = 0.2 ± 0.016 g) compared to their N-AMF-treated counterparts (Figure 3C,D). However, at the extreme salinity of 225 mM and 300 mM, the AMF inoculation did not show promising improvement in SDW and RDW. Results for the number of nodes showed that AMF treatment significantly improved the nodal formation of plants which was recorded as highest in the AMF-associated plants (5 ± 0.3 nodes/plant) under non-saline conditions followed by AMF-inoculated plants (4.6 ± 0.4 nodes/plant) at 75 mM NaCl (Figure 3). Conversely, when compared to the AMF-treated plants, the N-AMF plants showed more nodal formation at 225 mM NaCl stress, but this result was not significant.

**Figure 4:**
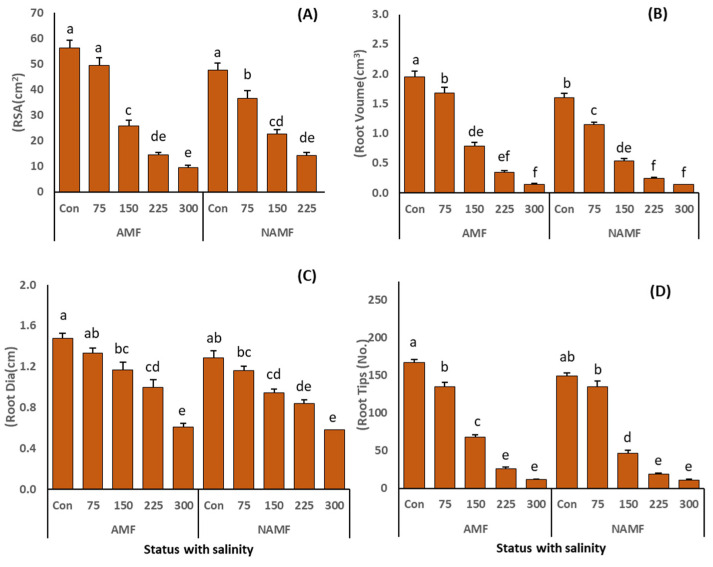
(**A**–**D**): indicates the role of AMF application as consortium (*C. etunicatum*, *F. mosseae*, *R. fasciculatum*, and *R. intraradices*) on root indices such as (**A**) root surface area (RSA, cm^2^), (**B**) root volume (RV, cm^3^), (**C**) root diameter (RD, cm), and (**D**) number of roots (RT, no/plant) of *C. ciliaris* plants grown under salinity stress. The results are presented as a mean ± standard error of five replicates. Different letters on top of the bars indicate the significant difference in treatments *p* = 0.05 (Tukey’s HSD test).

The results for number of leaves showed the same trend as that of other parameters where AMF inoculation induced a significantly promising improvement. Figure 4A–D displays the results of root indices such as RSA, RV, RD and RT. The increasing salinity stress significantly reduced all these parameters (Table 2). However, the application of AMF as a consortium of different species incorporated a marked improvement at all the salinity levels. The highest increase in root indices was recorded in AMF-inoculated plants (RSA = 56.3 ± 2.97 cm^2^, RV = 1.9 ± 0.09 cm^3^, RD = 1.5 ± 0.05 cm, and RT = 166.6 ± 4.8) under non-saline conditions which was followed by AMF-associated plants grown at 75 mM NaCl.

### 3.3. Impact of Inoculation with AMF in Consortium on Chlorophyll Content of C. ciliaris Grown under Salt Stress

**Figure 5:**
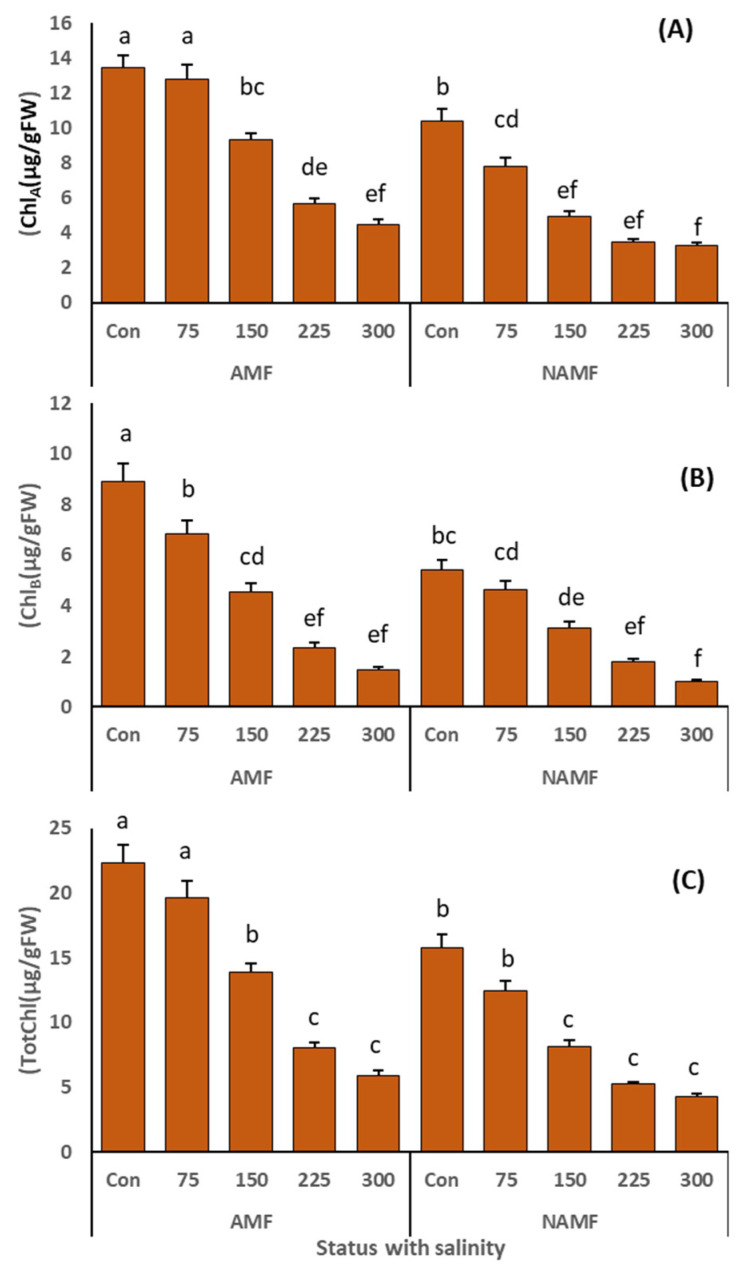
(**A**–**C**): indicates the role of AMF application as a consortium (*C. etunicatum*, *F. mosseae*, *R. fasciculatum*, and *R. intraradices*) on the photosynthetic pigment concentration, such as (**A**) chlorophyll *_a_* (µg/gFW), (**B**) chlorophyll *_b_* (µg/gFW) and (**C**) total chlorophyll (µg/gFW) of *C. ciliaris* plants grown under salinity stress. The results are presented as a mean ± standard error of five replicates. Different letters on top of the bars indicate the significant difference in treatments *p* = 0.05 (Tukey’s HSD test).

Increasing salinity stress significantly deteriorated the photosynthetic pigments (chlorophyll *_a_*, chlorophyll *_b_* and total chlorophyll) in both AMF and N-AMF-inoculated plants (Figure 5A–C, Table 3). However, the application of AMF alleviated the effects of salinity to a significant extent. Chlorophyll *_a_* (13.45 ± 0.68 µg/g fresh weight leaf), chlorophyll *_b_* (8.9 ± 0.7 µg/g fresh weight leaf) and total chlorophyll (22.4 ± 1.3 µg/g fresh weight leaf) were highest in the AMF inoculated plants under non-saline conditions followed by the AMF inoculated plants grown under 75 mM salinity stress.

### 3.4. Impact of Inoculation with AMF in Consortium on Proline and Phenolic Content of C. ciliaris Grown under Salt Stress

**Figure 6:**
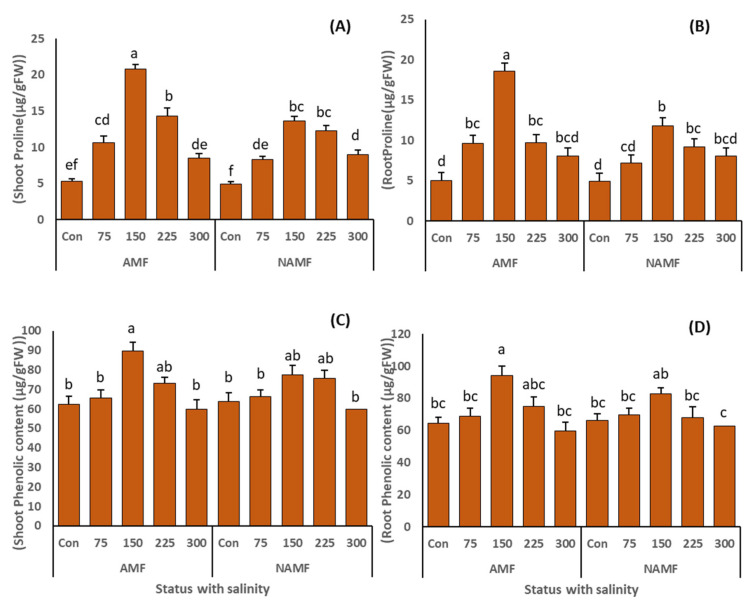
(**A**–**D**) indicates the role of AMF application as a consortium (*C. etunicatum*, *F. mosseae*, *R. fasciculatum*, and *R. intraradices*) on the proline and phenolic content (**A**) proline shoot (µg/gFW), (**B**) proline root (µg/gFW), (**C**) phenolic content shoot (µg/gFW) and (**D**) phenolic content root (µg/gFW) in *C. ciliaris* plants grown under salinity stress. The results are presented as a mean ± standard error of five replicates. Different letters on top of the bars indicate the significant difference in treatments *p* = 0.05 (Tukey’s HSD test).

The results of root and shoot proline content of *C. ciliaris* plants grown under saline stress in AMF- and N-AMF-amended soils are shown in Figure 6A,B. The shoot and root proline content of the plants inoculated with AMF and N-AMF showed a significant accumulation at the first three salinity stress levels. However, the proline buildup at 150 mM NaCl was exponential, which showed a gradual decline at the extreme salinity stress of 300 mM NaCl (Table 3). Moreover, the increase in proline accumulation under salinity application was significantly enhanced by the AMF inoculation in both shoot and root systems. The highest proline accumulation of 20.8 ± 0.58 µg/gFW and 18.5 ± 1.38 µg/gFW were recorded in AMF-inoculated plants at 150 mM salinity stress for shoot and root, respectively.

The results for phenolic content showed a similar trend as that of proline (Figure 6C,D). Application of NaCl stress induced the accumulation of total phenolic content for both root and shoot (Table 3). The highest production of phenolics was witnessed in the plants grown at 150 mM salinity stress, and showed a gradual decline at the extreme salinity stress of 300 mM. The production of phenolic compounds was further enhanced by the application of AMF as all AMF-treated plants displayed more phenol accumulation than the N-AMF-treated plants. The highest phenolic content (89.6 ± 4.5 µg/g shoot fresh weight) for shoot and (93.95 ± 5.8 µg/g root fresh weight) for root was recorded in AMF-inoculated plants at 150 mM NaCl stress.

## 4. Discussion

Soil salinity is considered as one of the most common abiotic stresses that produce deleterious impacts on the growth of plants [2,48] through osmotic stress and ion toxicity [12,23]. Moreover, it has become a major contributor to land degradation globally [49]. AMF, through symbiotic association with plants and thereby imparting enhanced salinity ameliorating mechanisms, have been linked to boosting plant growth, photosynthesis, and tolerance of salinity stresses [2,23]. In this experiment, we studied the effects of AMF application on the growth of *C. ciliaris* plants through improved photosynthetic pigment accumulation as well as enhanced proline and phenolic production under salinity stress.

In the present study, the *C. ciliaris* roots were colonized by AMF under all the salinity levels (Figure 2). This behavior of AMF is in harmony with previous studies where the plants were easily colonized by AMF under saline conditions [2,50]. The microscopic study of roots depicted the presence of all the colonization structures, viz., mycelium, vesicles, and arbuscules (Figure 2). However, the rate of colonization was significantly reduced by the increasing salinity. Moreover, our results depicted a significant decline in AMF sporulation under increasing NaCl application (Figure 2). These results are in accordance with previous studies wherein high levels of salt in the soil hindered spore germination, impeded hyphal growth, decreased the production of vesicles and arbuscules and hampered sporulation [51,52]. The increase in mycelial and arbuscular formation in the roots of *C. ciliaris* at the salinity levels of 75 mM and 150 mM could be because the salinity stress induced the speedy formation of these colonization structures as an adaptive measure for AMF to complete its life cycle [53]. Additionally, our observations align with the theory that AMF spend most of their resources on storage capacity in the roots under stressed conditions [54]. These results are supported by previous studies [2,55] and explain the improved growth of plants under salinity stress.

AMF symbiosis is well known to ameliorate salinity stress and improve plant growth which is also documented in various studies [2,56]. Results of this study revealed that the application of salinity deleteriously affected the growth parameters, such as SH, RL, SDW, RDW, RSA, RV, RD, RT, number of nodes and leaves, of *C. ciliaris* plants (Figure 3 and Figure 4). However, the application of AMF alleviated the negative effects of salinity and induced improvement in all the studied morphological indices. These findings are in line with the studies of [2,57] where the AMF inoculation induced an increase in the shoot/root morphological indices of *Lasiurus scindicus* L. and *Citrillus lanatus* L. under salinity stress. The increase in plant growth indices produced via AMF symbiosis is linked to the procurement of nutrients [53], more water uptake [58], P absorption [59], and improved osmotic potential of soil [60], all of which contribute to the morphological growth of the plant. Moreover, the hyphal network of AMF associated with plants can retain Na^+^ ions and inhibit their translocation to the shoot system and maintain the balance of K^+^/Na^+^ ratio, thereby helping the plants to sustain better growth than N-AMF plants [61]. Furthermore, a better root system (including improved RSA, RV, RD, and RT) reflects the morphological plasticity of plants under salinity stress [62], which is further heightened by AMF colonization [63]. The improved root architecture resulting from AMF symbiosis enables plants to absorb water and nutrients more efficiently, thereby increasing their adaptability to saline environments [64].

In salinity stress, chlorophyll reduction has long been thought to be a marker of oxidative stress during chlorophyll production [65,66] and linked to improved chlorophyllase activity that has a role in the decline of 5-aminolaevulinic acid (ALA) accumulation (a precursor of protochlorophyllide which converts to chlorophyll under sunlight) [67] which leads to declines in photosynthesis and the growth of plants [10,68,69]. The application of salinity in this study caused a certain decline in the metabolism of photosynthetic pigments, and hence growth (Figure 5). However, plants associated with AMF showed improved photosynthetic activity (higher chlorophyll *_a_*_, *b*_ and total chlorophyll contents) which was correlated with the enhanced growth of *C. ciliaris* under salinity stress. These results were in line with [70] for *Zea mays* L., and [71] for *Solanum lycopersicum*. Furthermore, in *Lasiurus scindicus* [2] found that AMF inoculation enhanced chlorophyll content in non-saline and saline conditions. AM symbiosis might raise not only the Mg intake [51] but also release hormonal signals that promote the formation of chloroplasts [72]. Therefore, it is evident from our findings that inoculation with AMF increases chlorophyll contents and alleviates the adverse salt stress effect to a certain extent.

Proline is one of the vital osmolytes which regulates salinity stress effectively in plant species via osmotic adjustment [73], keeps macromolecules safe during dehydration [74], acts as a hydroxyl radical scavenger [75], retains optimal NADP+/NADPH ratios, supports the stabilization of membrane proteins, lipids and other cellular structures [76], and serves as a critical energy source for plants that are exposed to saline conditions [77]. In our study, salt stress induced proline accumulation in both the root and shoot systems of *C. ciliaris* plants, which was further enhanced by AMF amendment (Figure 6A,B). The overproduction of proline in AMF-treated *C. ciliaris* could also explain its improved growth as it protects the plants from the damage caused by higher levels of salt-induced ROS [2]. Our investigation outcomes regarding proline accumulation in AMF-inoculated plants were in line with the findings of [60] in *Cucumis sativus*, [78] in *Ocimum basilicum*, and [79] in *Phragmites australis*. One explanation among others for the increased accumulation of proline in AMF-inoculated plants is that the initiation of root colonization by AMF triggers the defense system set up by the plant, which initially perceives AMF as stress or an attack. One possible reason among others for the higher proline buildup in AMF-inoculated plants is that early root colonization by AMF activates the plant’s defense system, as the plant perceives AMF as a stressor or threat which leads to the production of secondary metabolites [80].

Further, the results for phenolic content accumulation in our study showed an upward curve under salt stress (Figure 6C,D). These results were in line with the findings of [81,82]. Phenolics comprise numerous secondary metabolite groups, which perform many crucial functions, such as defense against various abiotic stresses [83]. Salinity stress induces the increased accumulation of enzyme phenylalanine ammonia-lyase (PAL; EC 4.3.1.5), which plays a crucial role in the production of cinnamic acid—a key component in the synthesis of phenolic compounds [84]. Furthermore, under salt stress, the nonenzymatic antioxidants integrated with phenolic compounds help the plants in scavenging toxic radicals [85]. The overproduction of phenolic compounds in our study was further improved by applying AMF on *C. ciliaris* plants growing under salinity stress. These results are consistent with those of [2,60].

## 5. Conclusions

In conclusion, this study shows that AMF inoculation (consortium of *C. etunicatum*, *F. mosseae*, *R. fasciculatum*, and *R. intraradices*) significantly reduced the adverse effects of salinity stress on *C. ciliaris*, thereby enhancing its growth by boosting its physiological traits and photosynthetic activity. Results also showed that AMF symbiosis increased the level of photosynthetic pigments, proline, and phenolics, which were otherwise reduced by salinity. This approach has significant potential for improving restoration efforts in salinity-affected ecosystems. However, further research involving diverse AMF species and field conditions may be required to validate our findings, particularly in the open saline ranges.

## Figures and Tables

**Table 1:** pH, EC, N, P and K of the soil collected from Al-Ghat, Riyadh, Saudi Arabia.

Location	pH	EC (dS m^−1^)	K (mg kg^−1^)	N (mg kg^−1^)	P (mg kg^−1^)	Texture
Al-Ghat	7.83	2.89	0.59	227.33	45.67	Sandy loam
	±0.03	±0.88	±0.075	±3.11	±14.13	

**Table 2:** Analysis of variance (Factorial design) of shoot and root morphological parameters in *C. ciliaris* treated with different levels of salinity under AMF and NON-AMF soil.

**Source of** **Variation**	**DF**	**SS**	**MS**	**F**	***p* Value**	**SS**	**MS**	**F**	***p***
		Shoot Length (cm)				Shoot Dry Mass (g)			
Status (A)	1	35.82	35.82	4.01	0.0527 *	0.0526	0.0526	26.43	<0.0001 ***
Salinity (B)	4	5932.07	1483.02	166.19	<0.0001 ***	1.897	0.474	234.94	<0.0001 ***
A × B	4	43.91	10.98	1.23	0.3153 ns	0.0462	0.011	5.8	0.001 **
		Nodes (No.)				Leaves (No.)			
Status (A)	1	4.5	4.5	11.84	0.001 **	4.5	4.5	7.14	0.011 *
Salinity (B)	4	74.52	18.63	49.03	<0.0001 ***	196.92	49.23	78.14	<0.0001 ***
A × B	4	3	0.75	1.97	0.119 ns	10.6	2.65	4.21	0.006 **
		Root Length (cm)				Root Dry Mass (g)			
Status (A)	1	900	900	10.47	0.002 **	0.022	0.022	11.11	0.002 **
Salinity (B)	4	90,173.9	22,543.5	262.14	<0.0001 ***	1.219	0.304	152.36	<0.0001 ***
A × B	4	1282.3	320.6	3.73	0.0123 *	0.013	0.003	1.65	0.183 ns
		RSA (cm^2^)				Root Volume (cm^3^)			
Status (A)	1	344.8	344.79	16.85	0.0002 ***	0.756	0.755	55.92	<0.0001 ***
Salinity (B)	4	13,503.4	3375.85	164.98	<0.0001 ***	20.197	5.049	373.54	<0.0001 ***
A × B	4	289.9	72.47	3.54	0.015 *	0.44	0.11	8.14	0.0001 ***
		Root Dia (cm)				Root Tips (No.)			
Status (A)	1	0.288	0.288	19.23	0.0001 ***	1076	1076.5	14.13	0.0006 ***
Salinity (B)	4	3.743	0.935	62.35	<0.0001 ***	174,884	43,720.9	573.83	<0.0001 ***
A × B	4	0.053	0.0133	0.89	0.480 ns	955	238.8	3.13	0.0261 *

RSA: Root Surface Area; *** *p* < 0.0001, ** *p* < 0.001, * *p* < 0.05, and “ns” as non-significant for *p* > 0.05.

**Table 3:** Analysis of variance (Factorial design) of biochemical parameters in *C. ciliaris* treated with different levels of salinity under AMF and NON-AMF soil.

**Source of Variation**	**DF**	**SS**	**MS**	**F**	***p* Value**	**SS**	**MS**	**F**	***p* Value**
		ChlA				ChlB			
Status (A)	1	126.14	126.144	105.16	<0.0001 ***	32.76	32.76	49.64	<0.0001 ***
Salinity (B)	4	494.05	135.51	102.97	<0.0001 ***	246.08	61.52	93.21	<0.0001 ***
A × B	4	24.179	6.045	5.04	0.0025 **	15.79	3.947	5.98	0.0009 ***
		Shoot Proline				Root Proline			
Status (A)	1	66.49	66.49	28.06	<0.0001 ***	49.2	49.2	13.91	0.0007 ***
Salinity (B)	4	854.49	213.62	90.16	<0.0001 ***	553.29	138.32	39.09	<0.0001 ***
A × B	4	88.81	22.2	9.37	<0.0001 ***	81.46	20.36	5.76	0.001 **
		Shoot Phenolics				Root Phenolics			
Status (A)	1	30.09	30.09	0.32	0.576 ns	73.35	73.35	0.64	0.430 ns
Salinity (B)	4	3707.95	926.98	9.78	<0.0001 ***	4372.4	1093.1	9.47	<0.0001 ***
A × B	4	362	90.5	0.96	0.443 ns	391.12	97.78	0.85	0.505 ns
		Mycelium				Vesicles			
Status (A)	1	32,207.2	32,207.2	1243.5	<0.0001 ***	4 × 10^6^	4274.93	200.02	<0.0001 ***
Salinity (B)	4	9173.1	2293.3	88.54	<0.0001 ***	1105.8	276.45	12.93	<0.0001 ***
A × B	4	9173.1	2293.54	88.54	<0.0001 ***	1105.8	276.45	12.93	<0.0001 ***
		Arbuscules				Total spore count			
Status (A)	1	19,391.1	19,391.1	487.26	<0.0001 ***	684,216	684,216	962.55	<0.0001 ***
Salinity (B)	4	7727.4	1931.8	48.54	<0.0001 ***	198,989	49,747	142.69	<0.0001 ***
A × B	4	7727.4	1931.8	48.54	<0.0001	198,989	49,747	142.69	<0.0001 ***

*** *p* < 0.0001, ** *p* < 0.001, and “ns” as non-significant for *p* > 0.05.

## Data Availability

Data is contained in the article.
